# Characterization of the Skeletal Muscle Proteome in Undernourished Old Rats

**DOI:** 10.3390/ijms23094762

**Published:** 2022-04-26

**Authors:** Caroline Barbé, Jérôme Salles, Christophe Chambon, Christophe Giraudet, Phelipe Sanchez, Véronique Patrac, Philippe Denis, Yves Boirie, Stéphane Walrand, Marine Gueugneau

**Affiliations:** 1 Human Nutrition Unit, INRAE, Auvergne Human Nutrition Research Center, Clermont Auvergne University, 63000 Clermont-Ferrand, France; caroline.barbe@uca.fr (C.B.); jerome.salles@inrae.fr (J.S.); christophe.giraudet@inrae.fr (C.G.); phelipe.sanchez@inrae.fr (P.S.); veronique.patrac@inrae.fr (V.P.); philippe.denis.2@inrae.fr (P.D.); yves.boirie@inrae.fr (Y.B.); stephane.walrand@inrae.fr (S.W.); 2 Animal Products Quality Unit (QuaPA), INRAE, 63122 Clermont-Ferrand, France; christophe.chambon@inrae.fr; 3 Metabolomic and Proteomic Exploration Facility, Clermont Auvergne University, INRAE, 63122 Clermont-Ferrand, France; 4 Department of Clinical Nutrition, Clermont-Ferrand University Hospital Center, 63000 Clermont-Ferrand, France

**Keywords:** skeletal muscle, aging, sarcopenia, undernutrition, proteome

## Abstract

Aging is associated with a progressive loss of skeletal muscle mass and function termed sarcopenia. Various metabolic alterations that occur with aging also increase the risk of undernutrition, which can worsen age-related sarcopenia. However, the impact of undernutrition on aged skeletal muscle remains largely under-researched. To build a deeper understanding of the cellular and molecular mechanisms underlying age-related sarcopenia, we characterized the undernutrition-induced changes in the skeletal muscle proteome in old rats. For this study, 20-month-old male rats were fed 50% or 100% of their spontaneous intake for 12 weeks, and proteomic analysis was performed on both slow- and fast-twitch muscles. Proteomic profiling of undernourished aged skeletal muscle revealed that undernutrition has profound effects on muscle proteome independently of its effect on muscle mass. Undernutrition-induced changes in muscle proteome appear to be muscle-type-specific: slow-twitch muscle showed a broad pattern of differential expression in proteins important for energy metabolism, whereas fast-twitch muscle mainly showed changes in protein turnover between undernourished and control rats. This first proteomic analysis of undernourished aged skeletal muscle provides new molecular-level insight to explain phenotypic changes in undernourished aged muscle. We anticipate this work as a starting point to define new biomarkers associated with undernutrition-induced muscle loss in the elderly.

## 1. Introduction

Undernutrition is an imbalanced nutritional state resulting from a negative ratio between dietary intake and nutrient needs. This imbalance may be related to inadequate intake of dietary energy and/or protein, an increase in energy expenditure and/or protein loss, or both [1]. Prolonged undernutrition leads to altered cellular metabolism and impaired body composition and function, which worsen disease prognosis [2]. In particular, undernutrition induces a loss of body tissues, especially a loss of skeletal muscle mass, which makes it one of the main risk factors for the onset of sarcopenia. Sarcopenia is defined as an independent disease entity related to a loss of muscle mass, quality, and strength [3,4]. Indeed, preserving skeletal muscle is not only crucial for maintaining mobility, but also for its many roles in metabolism and homeostasis. Low skeletal muscle mass has been shown to adversely affect health outcomes and lead to increased risk for mortality [5].

Aging increases the body’s needs for nutrients, especially protein intake. Older people show higher splanchnic extraction of amino acids [6,7] and lower anabolic response to protein diet [8], whereas various aging-related impairments lead to lower food intake such as poor dentition, physical and mental disabilities, multiple medications, and even social and financial challenges [9]. Undernutrition is therefore common in the elderly where its prevalence varies from 4% to 10% at home and from 15% to 30% in care homes, and even reaches 30% to 70% in hospitals, depending on the diagnostic criteria used [10]. Most of all, aging impairs the ability to adapt to undernutrition, leading to severe loss of body weight and muscle [11,12,13,14]. Old rats show defective adaptation to long-term 50% dietary restriction initiated at an advanced age, resulting in dramatic body weight loss and deregulation of protein metabolism in terms of nitrogen balance and protein content, especially at the skeletal muscle level [12]. Undernutrition in elderly patients consequently exacerbates their state of frailty and dependence, thus greatly increasing the risk for morbidity and mortality [15]. This issue will become increasingly critical given the worldwide pattern of population aging. According to United Nations statistics on demographics and aging, the proportion of persons aged 65 or over in the world is projected to grow from 9% in 2019 to nearly 16% in 2050 [16]. Specific studies are needed to better understand the undernutrition-induced alterations in skeletal muscle in order to identify new therapeutic targets and prevent sarcopenia in the elderly population.

Few studies have investigated the impact of undernutrition on aged skeletal muscle, and the mechanisms responsible for muscle wasting in this context remain unclear. Previous work has highlighted a reduction in protein turnover and mitochondrial oxidative capacities in fast-twitch plantaris muscle [17,18]. Aged undernourished rats show blunted rates of muscle protein synthesis together with increased indexes of muscle proteolysis [18]. Moreover, the energy deficit resulting from undernutrition was associated with a decrease in mitochondrial density and function in plantaris muscle [18,19]. Due to their fiber type composition, skeletal muscles vary in their functional and metabolic properties and in their response to atrophying conditions [20]. However, undernutrition-related muscle alterations have never been investigated in aged slow-twitch muscles.

Nowadays, the progresses in protein separation and analysis techniques as well as in bioinformatics allow for the identification of thousands of proteins in a short time. Since the development of so-called “soft” ionization sources, MALDI and electrospray, mass spectrometry has grown considerably in the field of biology [21]. In this regard, the application of proteomics to study muscle atrophy have provided new insight into the muscle proteome remodeling under various catabolic conditions such as unloading [22], cancer cachexia [23], and aging [24]. However, none of them investigated the muscle proteomic changes associated with undernutrition. Here, we used a shotgun proteomic approach to characterize the cellular and molecular mechanisms responsible for muscle alterations in undernourished old rats. Our proteomic workflow included a fractionation step to improve the detection of low-abundance proteins, and we performed a comparison between the proteome of fast-twitch and slow-twitch muscles to reveal muscle type-specific changes. This proteomic profiling of undernourished aged skeletal muscle revealed that undernutrition has profound effects on muscle proteome, independently of its effect on muscle mass. Undernutrition-induced changes in muscle proteome appear to be muscle-type-specific: slow-twitch muscle showed a broad pattern of differential expression in proteins important for energy metabolism, whereas fast-twitch muscle mainly showed changes in protein turnover between undernourished and control rats. This study ultimately identified new potential muscle biomarkers of undernutrition in the elderly.

## 2. Results

### 2.1. Undernutrition Affects Muscle Mass and Function in Old Rats

**Figure 1:**
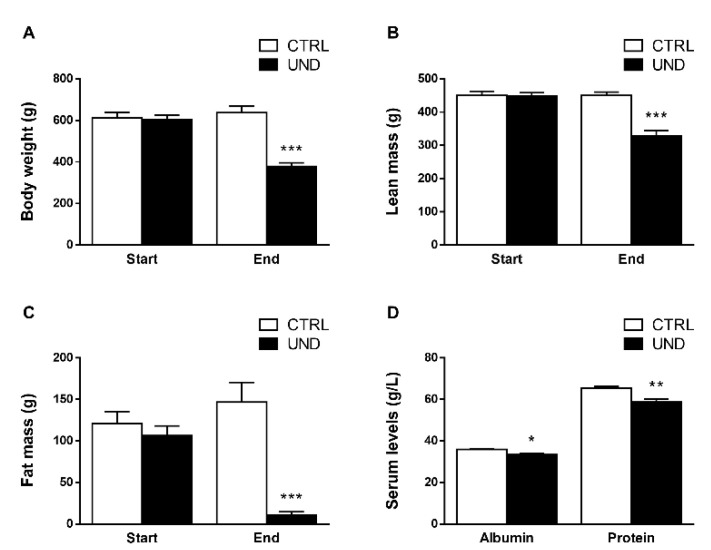
Body composition and nutritional markers in undernourished old rats. (**A**) Body weight, (**B**) EchoMRI-measured lean mass, (**C**) EchoMRI-measured fat mass, and (**D**) serum levels of nutritional markers from UND and CTRL old rats (n = 8–9/group). Results are expressed as means ± SEM. Unpaired student’s *t*-test were performed to test the effect of undernutrition (*, *P* < 0.05; **, *P* < 0.01 and ***, *P* < 0.001 vs. CTRL).

At the beginning of the experiment, there were no significant differences in body weight and body composition between the undernourished (UND) and control (CTRL) rats. At the end of the 12 weeks, we observed a decrease in body weight in response to undernutrition (−42% vs. CTRL, *P* < 0.001; Figure 1A) associated with a decrease in lean mass (−27% vs. CTRL, *P* < 0.001; Figure 1B) and a severe decrease in fat mass (−92% vs. CTRL, *P* < 0.001; Figure 1C). Nutritional status was also evaluated and revealed a slight decrease in serum albumin (−5%, *P* < 0.05) and total protein levels (−8%, *P* < 0.05) in UND compared to CTRL animals (Figure 1D), thus confirming undernutrition in the old rats.

**Figure 2:**
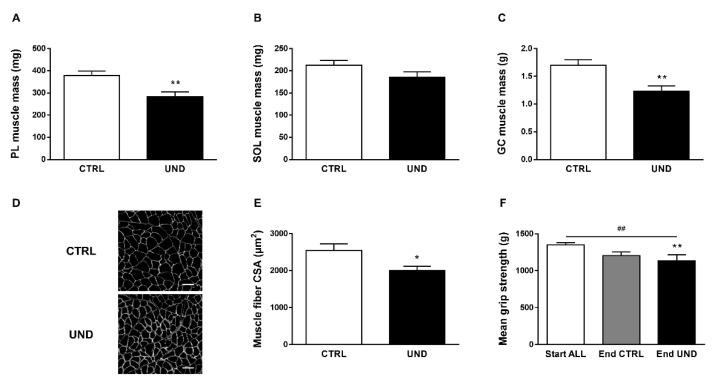
Muscle mass and function in undernourished old rats. (**A**) PL, (**B**) SOL, (**C**) GC muscle mass, (**D**) Representative images and (**E**) measurements of GC muscle fiber CSA (scale bar = 50 µm), and (**F**) forelimb mean grip strength in UND and CTRL old rats (n = 7–9/group). Results are expressed as means ± SEM. Unpaired student’s *t*-test was performed to test the effect of undernutrition (*, *P* < 0.05 and ** *P* < 0.01 vs. CTRL). Statistical analysis of mean grip strength used one-way ANOVA (^##^, *P* < 0.01) and Tukey post hoc tests (UND effect: ** *P* < 0.01).

In parallel, we observed a significant decrease in muscle mass, especially at hindlimb level including *Tibialis anterior* (TA), *Quadriceps* (QD), *Gastrocnemius* (GC), *Soleus* (SOL), and *Plantaris* (PL) muscles (9.27 ± 0.77 g in UND vs. 12.20 ± 0.63 g in CTRL; *P* < 0.01). In line with previous data [25], undernutrition led to a significant decrease in muscle mass in the predominantly fast-twitch PL muscle (−26% vs. CTRL; *P* < 0.01; Figure 2A) whereas the slow-twitch SOL muscle showed no significant difference (Figure 2B). Moreover, undernutrition-induced muscle atrophy was demonstrated by a decrease in GC muscle mass (−27% vs. CTRL; *P* < 0.01; Figure 2C) and fiber cross-sectional area (CSA) (−22% vs. CTRL; *P* < 0.05; Figure 2D). Regarding muscle function, whereas aging alone tends to decrease grip strength, UND further worsened the loss of absolute muscle force in old rats (−16% vs. Start; *P* < 0.05; Figure 2E).

### 2.2. Undernutrition Induces Profound Changes in Muscle Proteome Independently of Muscle Mass

**Figure 3:**
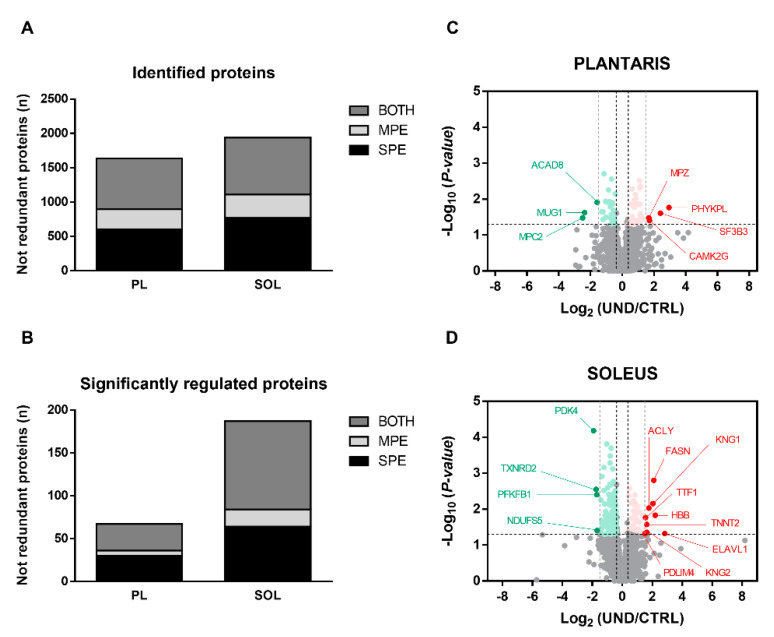
Undernutrition affects muscle proteome independently of its effect on muscle mass. (**A**) Total number of non-redundant proteins identified with at least two single peptides and (**B**) significantly regulated proteins between UND and CTRL PL and SOL muscles (*P* < 0.05 and 1.3-FC cut-off; n = 6/group). Volcano plots of the distribution of the 1635 and 1938 non-redundant proteins identified in (**C**) PL and (**D**) SOL muscles, respectively. *Y*-axis plots −Log_10_ (*P*-value) with a horizontal line at the significance threshold at *P* = 0.05. *X*-axis plots Log_2_ (UND/CTRL) with vertical black cut-off lines at +1.3-FC and −1.3-FC and vertical grey lines corresponding to +2.8-FC and −2.8-FC. Significantly downregulated and upregulated proteins are highlighted in green and red, respectively.

In order to better understand how undernutrition affects muscle mass and function in older age, we performed a shotgun analysis on both fast-twitch PL and slow-twitch SOL muscles. Given the wide dynamic range of protein expression in skeletal muscle, we used two-step solubilization of proteins and generated a sarcoplasmic protein-enriched extract (SPE) and a myofibrillar protein-enriched extract (MPE) fraction from each sample. Proteomic analysis was run on SPE and MPE separately. The amount of proteins extracted was similar in the SPE and MPE fractions of the non-atrophied SOL muscle between UND and CTRL rats (SPE: 34.41 ± 2.01 mg/g and 37.01 ± 1.39 mg/g, respectively, *NS*; MPE: 37.60 ± 2.53 mg/g and 38.07 ± 1.60 mg/g, respectively, *NS*). Conversely, muscle wasting was associated with a decrease in the amount of proteins extracted from PL muscle, specifically in the SPE fraction in UND rats compared to CTRL rats (SPE: 37.47 ± 0.91 mg/g and 43.41 ± 1.67 mg/g, respectively, *P* < 0.05; MPE: 84.21 ± 5.28 mg/g and 81.86 ± 6.59 mg/g, respectively, *NS*). Using this proteomic workflow, we identified a total of 1635 and 1938 non-redundant proteins with at least two single peptides in the PL and SOL muscles, respectively (FDR < 0.01). Around 38% of the proteins detected in SPE and 18% in MPE were identified, and 44% were identified in both protein extracts (Figure 3A). The differential analysis indicated that the abundance levels of 67 proteins in PL muscle (4% of the total detected proteins) and 187 proteins in SOL muscle (10% of the total detected proteins) were significantly altered in response to UND (Figure 3B) (*P* < 0.05 and 1.3-FC cut-off). The higher number of regulated proteins in the SOL muscle indicates that UND profoundly affects the muscle proteome, independently of its effect on muscle mass. As observed in the volcano plots, most of the differentially expressed proteins were downregulated in SOL muscle (75%), whereas PL had a relatively even proportion between upregulated and downregulated proteins (Figure 3C,D). However, a higher number of upregulated proteins showed extremely significant fold changes (±2.8-FC) between UND and CTRL rats, as annotated on the graphs.

**Figure 4:**
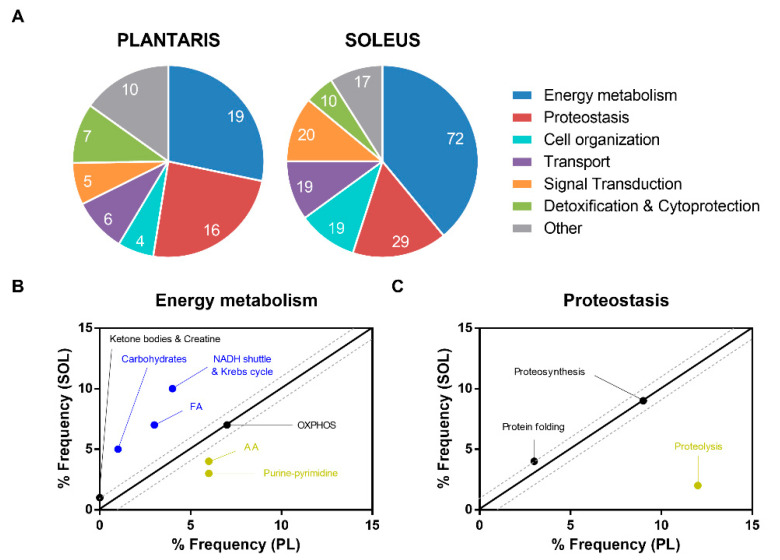
Functional annotation according to Biological Process enrichment analysis on the proteins dysregulated in PL and SOL muscles from undernourished rats. (**A**) Pie chart representing GO frequency within the dataset (i.e., 67 dysregulated proteins in PL muscle and 187 dysregulated proteins in SOL muscle (*P* < 0.05 and 1.3-FC cut-off; n = 6/group). Number of proteins dysregulated for each GO is reported directly on the graphs. The most over-represented GO_terms (**B**) “Energy metabolism” and (**C**) “Proteostasis” are scatterplotted, with blue/yellow enriched BP mainly found in SOL/PL muscle.

In order to identify the relevant biological processes (BP) involved in the muscle proteome changes observed in undernourished rats, we performed a Gene Ontology (GO) analysis on the significantly dysregulated proteins in PL and SOL muscles. The most over-represented GO_BP terms were “Energy metabolism” and “Proteostasis” in both PL and SOL muscle. We found that proteins involved in “Energy metabolism”, “Cell organization” and “Signal transduction” were more altered in the SOL than the PL muscle. Conversely, the proteins involved in “Proteostasis” and “Detoxification and Cytoprotection” were more altered in the PL muscle (Figure 4A).

Regarding metabolic processes, we found that carbohydrate and fatty acid (FA) metabolism and Krebs cycle were more altered in SOL than PL muscle whereas purine-pyrimidine and amino acid (AA) metabolism were more altered in PL than SOL muscle. There was no difference in regulation between the PL and SOL muscle in ketone bodies, creatine metabolism, and mitochondrial oxidative phosphorylation (OXPHOS) (Figure 4B). Regarding the proteostasis processes, proteolysis was more affected in PL than in SOL muscle whereas there was no difference between the PL and SOL muscles in proteosynthesis and protein folding (Figure 4C).

### 2.3. Regulation of Metabolism-Related Proteins in Muscle from Undernourished Old Rats

**Figure 5:**
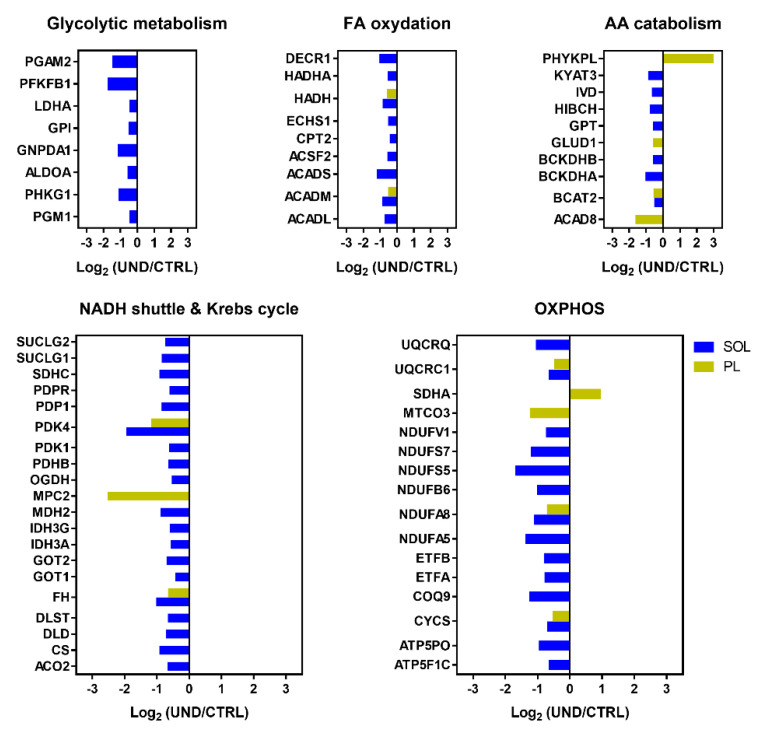
Regulation of metabolism-related proteins in muscle from undernourished old rats. Histograms of differential protein expression in the PL (yellow bars) and SOL (blue bars) muscles between UND and CTRL old rats (*P* < 0.05 and 1.3-FC cut-off; n = 6/group).

Our analysis showed that UND leads to a hypometabolic state in aged muscle, as highlighted by the decline in abundances of most of the metabolic enzymes, particularly at the mitochondrial level (Figure 5). Regarding glycolytic metabolism, we found that several glycogenolytic (PGM1, PHKG1) and glycolytic enzymes (ALDOA, GPI, LDHA, PGAM2, PFKB1) were less abundant in the SOL muscle from UND compared to CTRL rats.

Regarding muscle lipid metabolism, we observed a reduction in the abundance of proteins involved in FA beta-oxidation including medium-chain-specific acetyl-CoA dehydrogenase (ACADM) and hydroxyacyl-CoA dehydrogenase (HADH), which were found to be less abundant in both the PL and SOL muscles from UND compared to CTRL rats.

All the proteins involved in the Krebs cycle were found at lower levels in old UND rats including pyruvate dehydrogenase kinase isozyme 4 (PDK4) and fumarate hydratase (FH), which were decreased in response to UND in both the PL and SOL muscles. In SOL muscle, UND induced a decrease in the expression of enzymes responsible for the NADH shuttles (MDH2, GOT1 and GOT2). Moreover, our study points to lower levels of a large number of mitochondrial oxidative phosphorylation (OXPHOS) components in UND compared to CTRL rats including the complex I subunit NDUFA8, complex III subunit UQCRC1, and carrier protein cytochrome C (CYCS), transferring electrons from Complex III to Complex IV. These three proteins were less abundant in both the PL and SOL muscles. Surprisingly, the flavoprotein subunit of succinate dehydrogenase (SDHA), which is required for covalent FAD insertion into complex II of the mitochondrial electron transport chain and for the Krebs cycle, was significantly overrepresented in UND.

Finally, the decreased abundance of proteins involved in amino acid catabolism points to disruptions in amino acid metabolism, which contrasts with the decrease in muscle mass observed in response to UND. For example, branched-chain amino-acid aminotransferase (BCAT2) was less abundant in both the PL and SOL muscles from UND compared to the CTRL rats. This decrease in endogenous losses is likely to be secondary to the decrease in available energy.

### 2.4. Regulation of Proteostasis-Related Proteins in Muscle from Undernourished Old Rats

**Figure 6:**
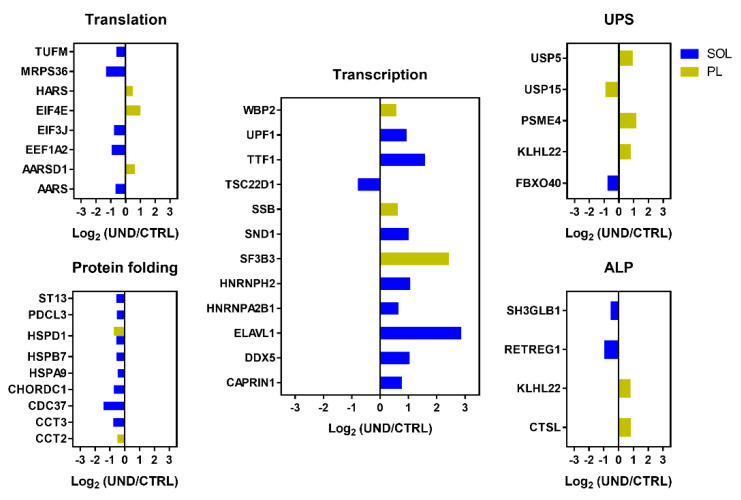
Regulation of proteostasis-related proteins in muscle from undernourished old rats. Histograms of differential protein expression in PL (yellow bars) and SOL (blue bars) muscle between UND and CTRL old rats (*P* < 0.05 and 1.3-FC cut-off; n = 6/group). UPS, ubiquitin–proteasome system; ALP, autophagy–lysosomal pathway.

Our proteomic data highlighted UND-induced alterations in protein synthesis and degradation (Figure 6). In line with the morphological changes, muscle protein turnover appeared to be differentially altered in PL vs. SOL muscles.

Regarding protein synthesis, PL muscle wasting was surprisingly associated with an increase in various transcription and translation regulators, and in particular, the ribosomal eukaryotic translation initiator factor 4E (EIF4E), which is a downstream effector of the mTOR pathway. Similar to PL muscle, SOL muscle showed a higher abundance of several transcriptional regulators and a lower abundance of the transcriptional repressor TSC22D1 protein. Unlike PL muscle, SOL muscle showed decreased levels of proteins involved in protein translation such as the initiation factors EIF3J and EEF1A2, the elongation factor TUFM, and the component of the 28S ribosomal subunit MRPS36. PL and SOL muscles both showed a decreased abundance of several chaperones involved in protein folding including HSPD1, which was downregulated in both PL and SOL muscles.

PL muscle wasting was associated with changes in the abundance of several ubiquitin–proteasome system (UPS) components. UND led to elevated levels of PSME4, an associated component of the proteasome. Although to date, only the deubiquitinating enzymes USP14 and USP19 have been found to be upregulated in atrophying muscle [26], here, we observed an increase in USP5 in response to UND in PL muscle. In contrast, USP15, which is involved in the regulation of hypertrophic response in cardiac muscle [27], was less abundant in PL muscles from UND compared to CTRL old rats. Alterations in the autophagy–lysosomal pathway (ALP) were revealed by an increase in the abundance of the lysosomal proteinase CTSL in response to UND. Moreover, KLHL22, which is an E3 ubiquitin ligase belonging to the BTB–CUL3–RBX1 E3 ubiquitin ligase complex (BCR) linking the UPS to autophagy termination [28], was more abundant in PL muscle from UND compared to CTRL old rats. Conversely, the E3 ubiquitin ligase FBXO40 targeting IRS1 [29] and the autophagy regulators SH3GLB1 and RETREG1 were found to be less abundant in the SOL muscle from UND old rats.

### 2.5. Proteomic Signature of Undernourished Aged Muscle

**Figure 7:**
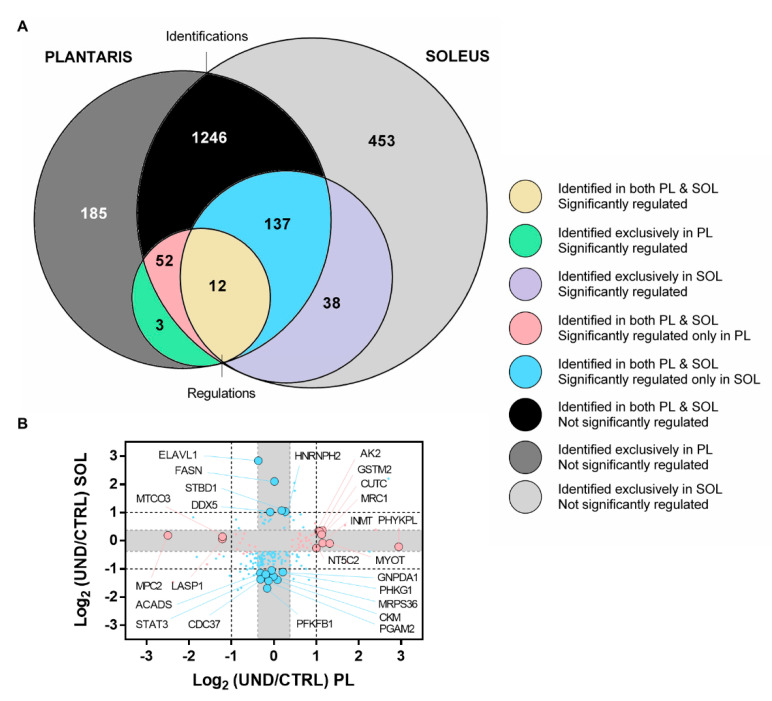
Comparison of proteomic changes in PL and SOL muscles from UND old rats. (**A**) Venn diagram comparing the number of identified and dysregulated proteins in the PL and SOL muscles from UND rats. (**B**) Scatterplot of Log_2_ (ratio UND/CTRL) for proteins identified in both PL and SOL muscles, but significantly regulated only in either the PL muscle (yellow dots) or SOL muscle (blue dots). The grey area indicates the 1.3-FC cut-off. Big dots highlight proteins specifically regulated at beyond +2-FC and −2-FC.

**Figure 8:**
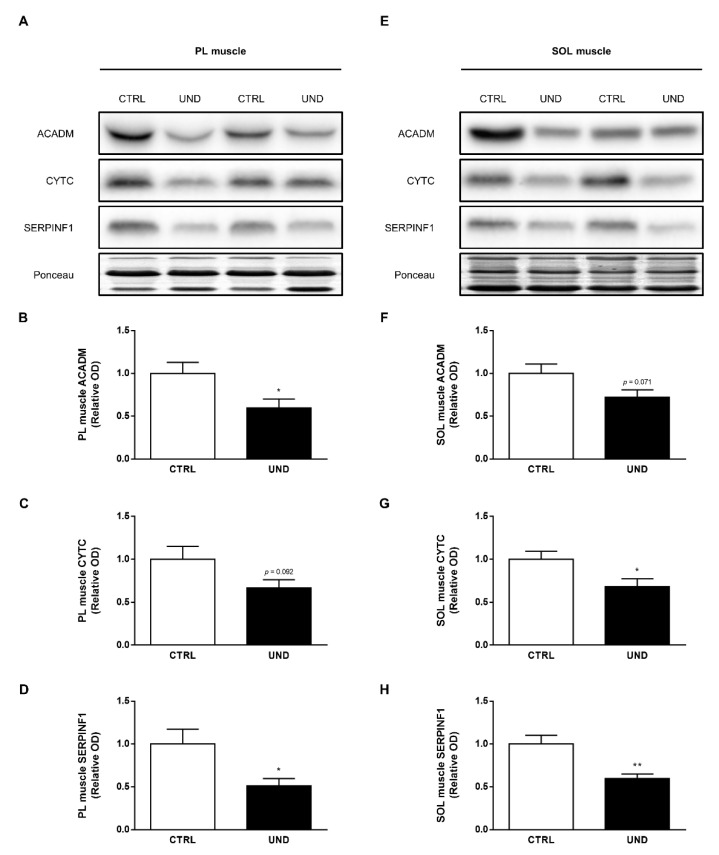
Western blot verification of selected muscle proteins commonly regulated by UND in both the PL and SOL muscles. (**A**,**E**) Representative western blot images and densitometric analysis of (**B**,**F**) β-oxidation enzyme ACADM, (**C**,**G**) oxidative phosphorylation enzyme CYTC, and (**D**,**H**) glycoprotein SERPINF1 levels in PL and SOL muscles from UND vs. CTRL rats (n = 6/group). Results are expressed as means ± SEM. Unpaired student’s *t*-test was performed to test the effect of undernutrition (*, *P* < 0.05; **, *P* < 0.01).

Comparative analyses showed that 1447 proteins were identified in both the PL and SOL muscle (Figure 7A). A total of 12 proteins were significantly regulated in the two muscles (Table 1), of which three were confirmed by western blotting (Figure 8). Note that for these proteins, directional changes were systematically concordant between the PL and SOL muscle. Moreover, three of these proteins (i.e., KNG2L, SERPINF1, and PDK4) were at least 2-fold differentially expressed in aged UND muscles, making them new potential muscle biomarkers of undernutrition in the elderly population.

In response to UND, the commonly-regulated protein with the biggest increase was KNG2L. Kininogens are plasma glycoproteins that have been linked to insulin sensitivity. Insulin sensitivity is reduced in kininogen-deficient rats [30]. Moreover, kinin, which results from kininogen cleavage by kallikrein, promotes an increase in insulin-stimulated glucose uptake in skeletal muscle [31,32,33]. In line with this observation, we observed a decrease in protein levels of SERPINF1, which is known to induce insulin resistance in skeletal muscle [34,35,36]. The pyruvate dehydrogenase kinase PDK4 was found to be decreased in skeletal muscle from old UND rats, whereas it increased in the skeletal muscle of insulin-resistant patients [37]. High-fat-diet-challenged PDK4 knockout mice exhibited improved glucose tolerance and insulin sensitivity [38,39]. Interestingly, it was recently shown that PDK4 overexpression is sufficient to induce C_2_C_12_ myotube atrophy whereas PDK4 blockade increases myotube size [40]. These regulations suggest that the decrease in insulinemia in response to UND [17] might be compensated for by increasing skeletal-muscle insulin sensitivity.

A large number of proteins were found to be significantly regulated in only one muscle (Figure 7A). In order to better visualize these proteins, we plotted their Log_2_ (UND/CTRL) values for PL muscle *versus* SOL muscle (Figure 7B). The number of muscle-specifically-regulated proteins ranged from 40 in the PL to 81 in the SOL muscle (cutoff < 1.3-fold), indicating that each muscle type exhibited a specific response to UND. As highlighted in Figure 7B, 11 of these proteins were at least 2-fold differentially expressed specifically in the PL muscle (Table 2) against 14 proteins in the SOL muscle (Table 3). In line with this observation, three proteins exclusively identified in PL muscle were at least 2-fold differentially expressed in response to UND (Figure 7A and Table 2). In parallel, 38 proteins exclusively identified in the SOL muscle were affected by UND (Figure 7A), of which 12 with at least 2-FC (Table 3). These fast- and slow-twitch proteins might therefore be responsible for the different responses of aged muscle to undernutrition.

Among the proteins specifically regulated in the fast-twitch PL muscle (Table 2), serpin family H member 1 (SERPINH1) and indolethylamine N-methyltransferase (INMT) have already been associated with muscle wasting conditions. Indeed, studies have reported a decrease in levels of SERPINH1, also known as HSP47, in response to hindlimb suspension and aging [41,42]. As SERPINH1 is a collagen specific molecular chaperone, its decrease suggests that undernutrition impedes maintenance of the extracellular matrix in skeletal muscle [43]. In contrast, gene expression of INMT has been found to be upregulated in response to hindlimb suspension and spaceflight [44].

Among the proteins specifically regulated in the slow-twitch SOL muscle (Table 3), several of the UND-increased proteins such as RRAS2 and STBD1 have already been associated with muscle hypertrophy. Indeed, activation of the canonical Ras/MAPK signaling has been shown to increase the activity of mTORC1, a crucial regulator of skeletal muscle hypertrophy [45]. Moreover, STBD1 is upregulated in muscle hypertrophy models induced by myostatin inhibition [46,47]. Although STBD1 has been linked to glycogen transport to lysosomes, its deletion does not affect glycogen content in skeletal muscle, suggesting an unknown function for this protein [48].

Moreover, we observed changes in the abundance of ELAVL1 and STAT3, which are activated in skeletal muscle and promote skeletal muscle atrophy in cancer [49]. The increased ELAVL1 level contrasts with the muscle phenotype observed in the SOL muscle. However, here, we found a lower abundance of STAT3, whereas STAT3 inhibition has been shown to abrogate skeletal muscle wasting in cancer cachexia models [50].

## 3. Discussion

Preserving skeletal muscle is not only crucial for maintaining mobility, but also for the many roles it plays in metabolism and homeostasis. Low muscle mass has been shown to adversely affect health outcomes and leads to increased risk for morbidity and mortality. Sarcopenia and undernutrition are both conditions that commonly occur in older populations, where they lead to weight loss with implications for muscle mass and strength and physical function [51]. Rapid worldwide population aging makes muscle health an increasingly real public health challenge. However, despite the co-existence of undernutrition and sarcopenia in older people, few studies have investigated the cellular and molecular mechanisms responsible for muscle wasting in undernourished old populations.

Previous research reported that 50% dietary restriction for 12 weeks was associated with a significant decrease in both fat mass and lean mass from old rats [17,18]. The UND protocol used here in old rats therefore logically affected the mass of several tissues including skeletal muscle. We found that UND had effects on the glycolytic PL muscle but had no significant effect on the oxidative SOL muscle. These findings are corroborated by previous data, demonstrating that UND does not affect muscle mass and protein synthesis in aged SOL muscle [17,25] and does not affect muscle mass and fiber CSA in adult SOL muscle [52]. Whether undernutrition affects the muscle fiber type composition remains undescribed. In addition, the mechanisms driving the differential effects of UND on these two muscle types are unclear. Interestingly, glycolytic fast-twitch muscle fibers show broader effects of aging than oxidative slow-twitch muscle fibers [6]. Moreover, it has been proposed that the preferential use of glucose as substrates might be rate-limiting for muscle protein synthesis in glycolytic muscles, making them more sensitive to UND [4]. In contrast, lipolysis-derived muscle FA might supply sufficient energy to ensure protein synthesis in oxidative muscles [7]. However, the differences in UND-induced alterations in fast and slow muscles remain largely unknown. To address this gap, we characterized changes occurring in skeletal muscle proteome from undernourished old rats and performed a shotgun analysis on both fast-twitch PL and slow-twitch SOL muscles. Our results demonstrate that, unlike muscle mass, UND affected the SOL muscle proteome more than the PL muscle proteome. Despite a similar number of identified proteins (SOL/PL ratio = 1.18), more proteins were found to be differentially expressed (SOL/PL ratio = 2.74) in the SOL muscle.

Undernutrition is often associated with muscle dysfunction and weakness in humans, and this was replicated here as a significant decrease in absolute muscle force in UND old rats. Muscle contraction is reliant on contractile proteins (i.e., myofibrillar proteins) and ATP supply, chiefly through mitochondrial oxidative function. Here, we did not find any change in myofibrillar protein content. However, our proteomic data revealed a reduced abundance in a large number of mitochondrial proteins, suggesting an alteration in ATP production. Grip strength test remains the measure of choice for assessing muscle strength in rodents, but it is influenced by many factors other than pure motor function [53]. Moreover, muscle function cannot be reduced to muscle strength, and in healthy older people, muscle power declines earlier and faster than muscle mass and strength [54]. Nevertheless, the decrease in muscle mass and grip strength observed here in response to UND suggests an alteration in global muscle function in these old rats.

Mitochondria are crucial for energy production, and the mitochondria organelle integrates several metabolic pathways. Our analysis showed a lower abundance of most of the mitochondrial proteins in response to UND, as highlighted by the reduced levels of several key enzymes in the Krebs cycle, β-oxidation, and OXPHOS complexes in both PL and SOL muscles. In line with this analysis, previous reports have shown altered mitochondrial function in the skeletal muscle of malnourished rats [17,18,52,55]. Our results indicate that alteration in mitochondrial function during UND is further complicated by the decrease in mitochondrial content. Moreover, the lower levels of β-oxidation enzymes were observed in SOL muscle while skeletal muscle mass was not significantly altered, which suggests that lipid oxidation may not provide sufficient energy to sustain protein synthesis in oxidative muscles. However, we also demonstrated that SOL muscle undergoes other proteomic changes that are likely to afford it better resistance to UND. Finally, the general decrease in the protein abundance of metabolic enzymes suggests a decrease in metabolic rate that likely contributes to energy saving. Here, UND led to a lower abundance of certain translation regulators in the SOL muscle, which is coherent with the fact that protein synthesis is a high-energy-consuming process [56]. Moreover, the pro-transcriptional regulations observed here might effectively reflect a compensatory response to a drop in translational regulation. In contrast, in the PL muscle, we observed regulations that would be expected to promote protein synthesis via transcription processes and translation processes, which does not fit with the muscle loss observed in response to UND. Moreover, previous research found a decrease in protein synthesis in PL muscle from undernourished old rats [17,18]. Although the abundance of a protein does not directly reflect its corresponding activity, these regulations could constitute or reflect important adaptations designed to limit the reduction in protein synthesis. Whether they represent mechanisms contributing to muscle sparing remains to be determined.

Muscle wasting may be the result of a decrease in protein synthesis and an increase in muscle protein breakdown, which in turn reflects the activation of two major pathways, the UPS pathway and the ALS pathway [57]. Our proteomic data revealed a number of regulations in proteolytic regulators at the level of both UPS and ALS. Most importantly, these regulations were perfectly in line with the changes in muscle mass and protein content observed in both PL and SOL muscles in response to UND. The changes observed in PL muscle are pro-proteolysis, with a higher abundance of proteins that activate UPS and ALS. These results are consistent with the increased MURF1 mRNA levels previously shown in PL muscle from UND rats [18]. Interestingly, cons-proteolysis regulations were observed in the SOL muscle. Taken together, these results suggest that UND-related muscle wasting is caused by increased protein degradation in the fast-twitch PL muscle while the decrease in proteolytic markers could slow the muscle loss in slow-twitch SOL muscle. The proteomic signature of aged UND muscle highlighted specific changes related to muscle type susceptibility. The workflow used here enabled us to identify new potential mediators of muscle wasting in fast-twitch muscle such as SERPINH1 or INMT. In contrast, the specific regulations observed in SOL muscle such as changes in levels of RRAS2, STBD1, and STAT3 could mediate the lower susceptibility of slow-twitch muscle to UND-induced muscle atrophy.

Many proteomic studies have investigated muscle aging in rodents and human skeletal muscle in order to characterize the molecular mechanisms of aging and identify biomarkers that could be targets for the prevention and treatment of sarcopenia [24,58,59,60]. Surprisingly, none assessed age-prevalent undernutrition. This study describes the first proteomic study of undernourished aged skeletal muscle to investigate the mechanisms by which undernutrition affects skeletal muscle in an old population. Our proteomic analysis identified a huge number of proteins whose expression was dysregulated in undernourished old skeletal muscle. We analyzed both the SOL muscle, which is mainly composed of slow-twitch fibers, and the PL muscle, which is mainly composed of fast-twitch fibers, to reveal muscle type-specific changes. Our data demonstrate that UND has profound effects on muscle proteome, independently of its effect on muscle mass. Changes observed in the abundance of metabolic enzymes appear to be consistent with a hypometabolic state, likely to support energy saving. Most importantly, proteomic changes in response to UND, and particularly protein turnover, appear to be specific to muscle type. Finally, we identified several proteins that likely drive the effect of UND in aged skeletal muscle. Future studies should examine whether and to what extent such targets could serve as potential new biomarkers of undernourished aged skeletal muscle and/or novel targets for therapeutic intervention.

## 4. Materials and Methods

### 4.1. Animals and Undernutrition Protocol

All animal and experimental procedures were performed in accordance with the Clermont-Ferrand University (France) IRB guidelines, and the study was approved by the local ethics committee (permission number10635-2017071711566890v2). Eighteen twenty-month-old male WISTAR rats were purchased from Janvier-Labs (Le Genest-St-Isle, France). All rats were from the same batch and bred under the same conditions throughout their lives. They were single-housed under controlled conditions of light (12 h/12 h light/dark cycle) and temperature (22 ± 2 °C). During the acclimatization period, the rats had access to standard chow pellets and water *ad libitum*, and their spontaneous intakes (21 g food/day) were assessed. At the start of the experiment, the undernourished animals (UND, n = 9) were fed 50% of their spontaneous intake for 12 weeks to induce undernutrition, while control animals (CTRL, n = 9) were fed *ad libitum* all throughout the experiment, as previously validated [18].

### 4.2. Whole Body Composition and Grip Strength Analysis

At the start and end of the experiment, the animals were placed in an EchoMRI-100 analyzer (Echo Medical Systems LLC, Houston, TX, USA) to determine fat and lean body mass (g). Mean and maximal muscle force were determined by a grip test of forelimb muscles. Grip strength was assessed using a commercially-available force gauge (Bioseb, Vitrolles, France) by the same investigator. The apparatus consisted of a metal bar connected to a force transducer. The rats were gently allowed to grasp the bar with their forepaws, then pulled back steadily until the bar was released down. Three successive measures were performed at 10 min intervals. Results are presented as the mean recorded force value.

### 4.3. Biological Sample Collection

At the end of the protocol, 16-h-fasted rats were weighed and anesthetized with isoflurane. Blood was collected from the abdominal aorta, then the animals were euthanized by cervical dislocation. Blood was allowed to clot for 15 min at room temperature, and serum was collected after centrifugation at 2000× *g* for 10 min at 4 °C. *Tibialis anterior* (TA), *Quadriceps* (QD), *Soleus* (SOL), and *Plantaris* (PL) muscles were then harvested, weighed, and immediately snap-frozen in liquid nitrogen. *Gastrocnemius* (GC) muscles were harvested, weighed, and promptly frozen in isopentane chilled by liquid nitrogen (−160 °C) for histological analyses. Biological samples were stored at −80 °C until processed.

### 4.4. Nutritional Status Analyses

Serum levels of albumin and protein were measured on a Konelab 20 biochemical analyzer (Thermo-Electron Corporation, Waltham, MA, USA).

### 4.5. Histological Analyses

Two serial transverse 10-μm-thick sections were obtained from each GC muscle at −18 °C using a cryostat and mounted on glass slides. Cross-sections were labelled with anti-laminin-α1 (1:20; #L9393, MerckMillipore, Molsheim, France) to outline the fibers and resolved with a secondary antibody conjugated to Alexa-Fluor 488 (Invitrogen, Cergy-Pontoise, France). Images were captured using a high-resolution digital camera coupled to an Axio Vert.A1 inverted microscope (Zeiss, Okerkochen, Germany) at a resolution of 0.645 μm/pixel. Four fields, each containing at least 200 fibers, were analyzed per muscle. Fiber cross sectional area (CSA) was determined for each fiber using ImageJ 1.47v image processing software (National Institutes of Health, Bethesda, MD, USA).

### 4.6. Muscle Protein Extraction

For the proteomic analysis, we used the PL and SOL muscles from six animals (those presenting the body mass and skeletal muscle mass the closest to the mean) per group. These muscles were homogenized (50 mg/mL) in ice-cold buffer containing 40 mM Tris (pH 7.0), 2 mM EDTA, and 10% protease inhibitor cocktail (#4693124001, MerckMillipore) using an Ultra-Turrax homogenizer (IKALabortechnik, Staufen, Germany). The sarcoplasmic protein-enriched extracts (SPE) were collected after centrifugation at 10,000× *g* for 10 min at 4 °C. The remaining pellets were resuspended in a buffer containing 50 mM Tris (pH 7.0) and 8 M urea, and incubated for 1 h on ice. The myofibrillar protein-enriched extracts (MPE) were then collected after centrifugation at 5000× *g* for 5 min at 4 °C. Protein concentrations of SPE and MPE were assessed using the BiCinchoninic acid (BCA) assay method (Pierce™ BCA Protein Assay Kit, ThermoFisher Scientific, Courtaboeuf, France) and the Bradford method (Protein Assay Dye Reagent Concentrate, Bio-Rad Laboratories, Hercules, CA, USA), respectively, according to the manufacturer’s instructions.

### 4.7. Nano-LC-MS/MS Analysis

Proteins (22.5 µg) from both the SPE and MPE fractions were concentrated into the 1D electrophoresis stacking gel (12% acrylamide), as described in [61,62]. Briefly, gels were stained in Coomassie brilliant blue G-250 solution, and excised lanes were reduced with dithiothreitol (DTT) and alkylated with iodoacetamide (IAA). Proteins were then digested by trypsin (ratio 1:50), and peptide mixtures were analyzed by online nanoflow liquid chromatography using an Ultimate 3000 system coupled to a HFX mass spectrometer (ThermoFisher Scientific) with a nanoelectrospray ion source. Each peptide fraction (n = 48, two fractions per muscle and six animals per group) was injected once into the LC−MS/MS instrument. In other words, we performed LC-MS/MS mass spectrometry analysis in simplicate on six biological replicates per group. The MS/MS spectra search was performed using Mascot (v2.5.1) and Peaks (v10) against the Uniprot Rattus norvegicus database (2019/07, 29,944 sequences). The following parameters were considered for the search: precursor mass tolerance of 10 ppm and fragment mass tolerance of 0.05 Da, a maximum of two missed cleavage sites of trypsin, carbamidomethylation (C), oxidation (M), and deamidation (NQ) set as variable modifications. The minimal peptide length was 5–7 amino acids. Protein identification was validated when at least two peptides originating from one protein showed a significant Mascot score with a false discovery rate (FDR) ≤1%. Label-free protein quantification was performed using Progenesis QI (Nonlinear Dynamics, Waters, Milford, MA). All unique validated peptides of an identified protein were included, and the total cumulative abundance was calculated by summing the abundances of all peptides allocated to the respective protein. The mass-spectrometry proteomics data were deposited to the ProteomeXchange Consortium via the PRIDE [63] partner repository with the dataset identifier PXD032044. Statistical analysis was performed using the “between subject design” option and *P*-values were calculated by a repeated-measures analysis of variance using the normalized abundances across all runs. The significate threshold was set at *P* < 0.05 and a 1.3-FC cut-off was applied. To complete the analysis, the proteomic results for SPE and MPE were gathered. In the case of duplication between both fractions, the amplitude of the changes is reported for the fraction where the protein was the most expected based on Progenesis QI confidence score and the known protein localization. Note that directional changes were concordant between the two fractions for >75% of the proteins detected in both fractions, and reported as differentially abundant between UND and CTRL rats.

### 4.8. Gene Ontology Analysis

Functional annotation was performed according to the Gene Ontology (GO) Biological Processes (BP) using the ProteINSIDE web service (http://www.proteinside.org accessed on 20 April 2021) [64]. The analysis was performed in *Rattus norvegicus,* and both muscles were considered independently. Benjamini–Hochberg (BH) adjusted *P*-values were used to establish lists of significantly-enriched pathways in each dataset compared to the whole genome. The GO_BP overview was carried out using only specifications associated with an adjusted *P*-value < 0.001 with a minimum of annotated proteins ≥2. Finally, annotations were completed manually based on UniProt (https://www.uniprot.org/ accessed on 20 April 2021), NCBI (https://www.ncbi.nlm.nih.gov/ accessed on 20 April 2021), and DAVID (https://david.ncifcrf.gov/ accessed on 20 April 2021) databases.

### 4.9. Western Blots

Equal amounts of proteins were separated by SDS-PAGE and transferred to PVDF membranes. Membranes were probed with the following primary antibodies: anti-ACADM (1:500; #GTX100488, Genetex, Irvine, CA, USA), anti-SERPINF1 (1:2000; #AF1149, R&D Systems, Minneapolis, MN, USA), and anti-Cytochrome C (1:500; #4272, Cell Signaling Technologies, Leiden, The Netherlands), then incubated with a horseradish peroxidase-coupled secondary antibody and developed using an enhanced chemiluminescence (ECL) western blotting substrate (ThermoFisher Scientific). Luminescence was visualized using a Fusion FX imaging system (Vilber Lourmat, Collegien, France) and quantified using ImageJ 1.47v image processing software. All results were normalized to total protein Ponceau staining.

### 4.10. Statistical Analysis

For statistical analysis not related to the proteomic datasets, results were expressed relative to CTRL condition and are presented as means ± standard error of the mean (SEM). An unpaired Student’s *t*-test was used to assess statistical differences amongst means. Statistical analysis of mean grip strength was performed using one-way ANOVA followed by Tukey post hoc tests. The significance threshold was set at *P* < 0.05. All statistical analyses were performed using GraphPad Prism 6 software (San Diego, CA, USA).

## Figures and Tables

**Table 1:** Proteins regulated by UND in both the PL and SOL muscle from old rats.

Proteins ( 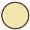 )		PL		SOL	
Symbol	Name	FC	*P*	FC	*P*
PDK4	Pyruvate dehydrogenase kinase isozyme 4	−2.2	0.002	−3.8	0.000
SERPINF1	Alpha-2 antiplasmin	−2.1	0.012	−2.1	0.000
NDUFA8	NADH dehydrogenase 1 alpha subcomplex subunit 8	−1.6	0.048	−2.1	0.015
HSPD1	60 kDa heat shock protein	−1.6	0.003	−1.4	0.012
FH	Fumarate hydratase	−1.5	0.014	−2.0	0.029
HADH	Hydroxyacyl-coenzyme A dehydrogenase	−1.5	0.028	−1.7	0.014
BCAT2	Branched-chain amino acid aminotransferase	−1.4	0.037	−1.4	0.023
CYCS	Cytochrome c, somatic	−1.4	0.047	−1.6	0.011
ACADM	Acetyl-coenzyme A dehydrogenase medium-chain	−1.4	0.040	−1.8	0.001
UQCRC1	Cytochrome b-c1 complex subunit 1	−1.4	0.040	−1.5	0.039
IMPDH2	Inosine-5′-monophosphate dehydrogenase 2	+1.7	0.045	+1.7	0.011
KNG2L	T-kininogen 2	+2.2	0.041	+3.1	0.044

**Table 2:** Proteins regulated by UND, specifically in PL muscle from old rats.

Proteins ( 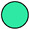 & 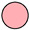 )		PL		SOL	
Symbol	Name	FC	*P*	FC	*P*
SERPINH1	Serpin H1	−2.0	0.031	ND	ND
CD163	Scavenger receptor cysteine-rich type 1 protein M130	+2.2	0.005	ND	ND
PSME4	Proteasome activator subunit 4	+2.2	0.014	ND	ND
MPC2	Mitochondrial pyruvate carrier 2	−5.7	0.033	+1.1	0.914
LASP1	LIM and SH3 domain protein 1	−2.3	0.037	+1.0	0.858
MTCO3	Cytochrome c oxidase subunit 3	−2.3	0.023	+1.1	0.602
NT5C2	5′-nucleotidase, cytosolic II	+2.0	0.040	−1.2	0.252
AK2	Adenylate kinase 2, mitochondrial	+2.1	0.045	+1.3	0.154
GSTM2	Glutathione *S*-transferase Mu 2	+2.1	0.003	+1.3	0.609
MRC1	Mannose receptor C-type 1	+2.2	0.028	+1.2	0.943
CUTC	CutC copper transporter	+2.2	0.004	+1.3	0.711
INMT	Indolethylamine N-methyltransferase	+2.2	0.025	−1.1	0.707
MYOT	Myotilin	+2.5	0.035	−1.1	0.538
PHYKPL	5-phosphohydroxy-L-lysine phospho-lyase	+7.7	0.017	−1.2	0.705

**Table 3:** Proteins regulated by UND specifically in the SOL muscle from old rats.

Proteins ( 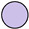 & 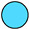 )		PL		SOL	
Symbol	Name	FC	*P*	FC	*P*
L2HGDH	L-2-hydroxyglutarate dehydrogenase	ND	ND	−2.1	0.015
AKAP1	A-kinase anchor protein 1	ND	ND	+2.0	0.036
ENPP3	Ectonucleotide pyrophosphatase/phosphodiesterase family member 3	ND	ND	+2.2	0.023
NOMO1	Nodal modulator 1	ND	ND	+2.2	0.012
UGDH	UDP-glucose 6-dehydrogenase	ND	ND	+2.3	0.040
CYP4B1	Cytochrome P450 4B1	ND	ND	+2.4	0.013
CHMP3	Charged multivesicular body protein 3	ND	ND	+2.6	0.017
RRAS2	RAS-related protein 2	ND	ND	+2.6	0.017
MXRA7	Matrix-remodeling-associated protein 7	ND	ND	+2.8	0.017
PDLIM4	PDZ and LIM domain protein 4	ND	ND	+2.9	0.048
TTF1	Transcription termination factor 1	ND	ND	+2.9	0.017
TNNT2	Troponin T, cardiac muscle	ND	ND	+3.1	0.027
PFKFB1	6-phosphofructo-2-kinase/fructose-2,6-biphosphatase 1	−1.1	0.719	−3.2	0.004
PGAM2	Phosphoglycerate mutase 2	−1.1	0.663	−2.7	0.006
CKM	Creatine kinase M-type	+1.1	0.868	−2.6	0.201
CDC37	Hsp90 co-chaperone Cdc37	−1.2	0.427	−2.6	0.034
MRPS36	Mitochondrial ribosomal protein S36	−1.0	0.760	−2.4	0.028
STAT3	Signal transducer and activator of transcription 3	−1.1	0.862	−2.3	0.050
ACADS	Acetyl-coenzyme A dehydrogenase, short chain	−1.2	0.113	−2.2	0.015
GNPDA1	Glucosamine-6-phosphate isomerase	+1.2	0.484	−2.2	0.016
PHKG1	Phosphorylase b kinase gamma catalytic chain, skeletal muscle/heart isoform	−1.1	0.978	−2.1	0.048
DDX5	DEAD (Asp-Glu-Ala-Asp) box polypeptide 5	−1.1	0.755	+2.0	0.008
HNRNPH2	Heterogeneous nuclear ribonucleoprotein H2	+1.2	0.837	+2.1	0.037
STBD1	Starch-binding domain-containing protein 1	+1.1	0.402	+2.1	0.049
FASN	Fatty acid synthase	+1.0	0.914	+4.3	0.002
ELAVL1	ELAV-like protein 1	−1.3	0.260	+7.2	0.047

## Data Availability

All data supporting the reported results can be found in the tables and figures. The mass spectrometry proteomic data were deposited at the ProteomeXchange Consortium via the PRIDE partner repository with the dataset identifier PXD032044.
